# Immunological alterations in patients with current and lifetime suicide ideation and attempts: Examining the relationship with depressive symptoms

**DOI:** 10.1016/j.bbih.2024.100777

**Published:** 2024-04-25

**Authors:** Leandro Nicolás Grendas, Eugenio Antonio Carrera Silva, Romina Isabel Álvarez Casiani, Alejandro Olaviaga, Josefina Robetto, Ángeles Romina Arena, Vera Tifner, Luciana Carla Chiapella, Marcelo Fisichella, Melina Bianca Penna, Fernando Hunter, Cintia Romina Prokopez, Andrea Emilse Errasti, Federico Manuel Daray

**Affiliations:** a Instituto de Farmacología, Facultad de Medicina, Universidad de Buenos Aires, Ciudad de Buenos Aires, Argentina; b Hospital General de Agudos “Dr. Teodoro Álvarez”, Ciudad de Buenos Aires, Argentina; c Instituto de Medicina Experimental (IMEX), Consejo Nacional de Investigaciones Científicas y Técnicas (CONICET), Academia Nacional de Medicina, Ciudad de Buenos Aires, Argentina; d Consejo Nacional de Investigaciones Científicas y Técnicas (CONICET), Argentina; e Hospital General de Agudos “Dr. Cosme Argerich”, Ciudad de Buenos Aires, Argentina

**Keywords:** Suicidal ideation, Suicidal attempts, Immune system, Inflammation, Immune cells

## Abstract

**Background:**

Suicidal ideation and attempt (SI/SA) have been associated with dysregulation of the immune response and inflammation. However, few studies have explored how innate and acquired cellular immunity impact on the peripheral immune response. Our study addresses this gap by examining the composition of peripheral immune cells and humoral markers among individuals with current SI/SA, individuals with a history of SI/SA, and healthy controls (HC). Additionally, we aim to explore whether depressive symptoms settle the relationship between inflammation and SI/SA.

**Methods:**

This is a multicenter case-control study that included 105 participants. Clinical and demographic characterists together with hemogram parameters, soluble pro and anti-inflamatory factors, and specific innate and adaptive immune cell populations were compared among patients with current SI/SA (n = 21), a history of lifetime SI/SA (n = 42), and HC (n = 42).

**Results:**

Patients with both current and lifetime SI/SA had a significant increase in the absolute count of monocytes and in the monocyte/lymphocyte ratio (MLR). Additionally, patients with current and lifetime SI/SA showed a significant increase in high-sensitivity C- reactive protein (hs-CRP), and patients with lifetime SI/SA also showed higher levels of Erythrocyte Sedimentation Rate (ESR). The cellular inflammatory status of patients with SI/SA was characterized by altered proportions of monocytes with higher levels of nonclassical and intermediate monocytes. No differences were observed in the number of lymphocytes and the proportion of CD4 and CD8 between patients and HC, but we found differences in markers of exhaustion of CD4 lymphocytes, with increased levels of Programmed cell death protein 1 (PD1) in Current SI/SA and Lymphocyte activation gene 3 (LAG3) in Current SI/SA and Lifetime SI/SA compared to HC. The plasmainflammatory status was marked by higher levels of soluble Triggering receptor expressed on myeloid cells 2 (sTREM2) in patients with lifetime SI/SA compared to HC. Finally, the multinomial analysis indicates that inflammation and depressive symptoms are independently associated with SI/SA.

**Conclusion:**

This study highlights the association of immunological alterations with SI/SA. Furthremore, SI/SA is independently influenced by depressive symptoms and inflammation. This may have important therapeutic implications, as in these patients, it may be necessary to treat the inflammatory process beyond treating the depressive symptoms.

## Highlights

• Alterations in peripheral immune cells are observed in patients with SI/SA.
• Patients exhibit increased monocytosis with an expansion of intermediate and nonclassical monocytes.
• An elevated percentage of CD4^+^CD25^+^FOXP3^+^ Tregs is also observed.
• Markers of T cell exhaustion of CD4 lymphocytes are detected in these patients.
• The relationship between inflammation and SI/SA is not moderated by depressive symptoms.

## 1 Introduction

Patients with suicidal ideation and attempt (SI/SA) have shown immune changes in blood, cerebrospinal fluid, and postmortem brain samples (Chang et al., 2016; Keaton et al., 2019; Sudol and Mann, 2017; Ducasse et al., 2015; Courtet et al., 2015; Serafini et al., 2020; González-Castro et al., 2021). These studies mainly focused only on the humoral component and showed increased peripheral C-reactive protein (Miola et al., 2021; Chen et al., 2020) and IL-6 levels (González-Castro et al., 2021) and decreased IL-1β levels (Ganança et al., 2021). While cells of the monocyte-macrophage lineage and T cells play crucial roles in immune surveillance in both the peripheral and central nervous system (Schiweck et al., 2020a, 2020b; Strazielle et al., 2016), the cell-mediated immune profile in patients with SI/SA remains poorly investigated (Schiweck et al., 2020b).

Most of the people who died by suicide appear to have had a psychiatric disorder at the time of death, being the affective disorders (AD) the most frequent cause (Cavanagh et al., 2003; Conwell et al., 1996; Hawton et al., 2013). It is also known that AD are a risk factor for SI/SA, and suicidality accounts a substantial proportion of the increased mortality observed in patients suffering from AD (Bachmann, 2018). Interestingly, a compelling body of evidence has emerged, indicating the potential involvement of the immune system in the development of AD. Patients with AD exhibit cardinal features of an inflammatory response, such as increased levels of pro-inflammatory cytokines and their receptors, along with the increased release of acute-phase reactants, chemokines, and soluble adhesion molecules in peripheral blood and cerebrospinal fluid (Miola et al., 2021; Berk et al., 2013; Miller et al., 2009; Miller and Raison, 2016; Enache et al., 2019; Long et al., 2024). It remains unclear whether the immune alterations in patients with AD and SI/SA are independent or not from depressive symptoms.

The present study has two aims. (1) To compare the composition of peripheral immune cells and humoral markers among individuals with current SI/SA, individuals with a history of SI/SA, and healthy controls (HC), and (2) to investigate whether the immunological changes in SI/SA patients correlate with depressive symptoms or remain independent of them.

## 2 Methods

### 2.1 Study design

The present study is a secondary analysis of data collected during the study “*Decoding the Inflammatory Signature of the Major Depressive Episode: Insights from Peripheral Immunophenotyping in Active and Remitted Condition a Case-Control Study*” (Daray et al., 2024a). The original research was a sex and aged-matched case-control multicentric study conducted between March 2019 and December 2022 in Buenos Aires, Argentina.

### 2.2 Sample

Patients were recruited from the *Hospital General de Agudos “Dr. Teodoro Álvarez”, Hospital General de Agudos “Dr. Enrique Tornú”, Hospital General de Agudos “Dr. Cosme Argerich”, Hospital General de Agudos “José María Ramos Mejía”*, and *Hospital Neuropsiquiátrico “Dr. Braulio A. Moyano”* in Buenos Aires. All these hospitals serve a sizable urban catchment area in Buenos Aires and treat mainly low-income patients without insurance. The Institutional Review Board of each Hospital approved the study.

For this secondary analysis, patients meeting the following criteria were included: (a) age between 18 and 65 years, (b) have current or history of suicide ideation (SI) or suicide attempt (SA), (c) diagnosed with DSM-5 Mayor Depressive Episode (MDE) either Major Depressive Disorder (MDD) or Bipolar Disorder, (d) willing and able to sign a consent form to participate. Exclusion criteria were: (a) have a comorbid diagnosis of obsessive-compulsive disorder (OCD), psychotic disorders, or posttraumatic stress disorder (PTSD), (c) a diagnosis of borderline personality disorder (BPD), or (d) diagnosis of substance use disorder within the past 30 days.

Healthy controls (HC) between 18 and 65 years were recruited ensuring a comparable sample. Exclusions for HC were: (a) having the diagnosis of any mental disorder, (b) having a diagnosis of substance use disorder within the past 30 days, (c) having a first-degree relative diagnosed with a mood disorder, d) not having the ability to sign a consent form.

Additional exclusion criteria for both the participants (SI/SA patients and HC) were (a) the presence of a chronic or acute physical illness with an inflammatory component, (b) receiving medication with anti-inflammatory or immunomodulatory properties, (c) getting infected with SARS-CoV-2 within the past 30 days before the evaluation, (d) having received the vaccine for SARS-CoV-2 or any other vaccine in the 30 days previous to the evaluation, (e) being pregnant, breastfeeding, having had an abortion or miscarriage during the previous 30 days to the evaluation.

### 2.3 Measures

A trained interviewer gathered information of participant characteristics, including questions regarding clinical and demographic variables. The diagnosis of Current SI/SA as well as Lifetime SI/SA, was determined by the Columbia-Suicide Severity Rating Scale (C-SSRS) (Posner et al., 2011). The International Neuropsychiatric Interview (MINI), version 7.0.2 (Sheehan et al., 1998), was used for diagnostic purposes. Based on these measures, three groups of participants were defined: Group 1: Current SI/SA (patients with SI/SA in the last month) and Group 2: Lifetime SI/SA (patients with a history of SI/SA at some point in their life before the previous month and without SI/SA in the last month). Group 3: Healthy Controls (HC) participants, did not present current SI/SA or lifetime SI/SA and did not meet any diagnostic criteria by the MINI for a psychiatric disorder.

Additionally, several other questionnaires were administered to control for potential sources of variation in inflammatory levels. Depression severity was assessed using the Hamilton Depression Rating Scale (HDRS-17) (Bobes et al., 2003). The Adverse Childhood Experiences (ACEs) questionnaire (Felitti et al., 1998), the Brugha Stressful Life Events Scale (Brugha and Cragg, 1990) and the International Physical Activity Questionnaire (IPAQ) (Craig et al., 2003) were also applied. Weight and height were also recorded.

### 2.4 Blood sample collection, processing, and biochemical analysis

Blood samples were drawn by venipuncture and collected into EDTA-coated tubes (BD, Vacutainer) in the morning on the same day of completing the psychiatric evaluations. A total of 20 mL of blood was obtained on the day of the clinical assessment. From these, 10 mL was used for routine biochemical laboratory tests, including the hemogram analysis, Erythrocyte Sedimentation Rate (ESR), and high-sensitivity C-reactive protein (hs-CRP) measurements. The remaining blood sample was used for direct immunophenotyping staining, plasma separation, and peripheral blood mononuclear cells (PBMC) isolation (Ortiz Wilczyñski et al., 2020).

### 2.5 Immunophenotyping by direct blood staining

To determine the circulating proportion of monocyte subsets, the activation and exhaustion markers on T cells, and the frequency of Tregs, three antibody panels were used as it was described by our group (Daray et al., 2024a). Briefly, we used the following combinations of antibodies (BioLegend) (1) **Monocytes panel**: CD11b-Brilliant Violet 421™ (Cat. 101251), HLA-DR-PE (Cat. 307606), CD86-biotin (Cat. 305404) plus DyLight™ 649-conjugated Streptavidin (Cat. 405224), CD14-PE/Cyanine7 (Cat. 325618), and CD16-fluorescein isothiocyanate (FITC) (Cat. 302005) (2) **T cell panel**: CD3-PE/Cyanine7 (Cat. 300316), CD4-APC/Cyanine7 (Cat. 317418), CD8-PE (Cat. 317418), CD69-PerCP/Cyanine5.5 (Cat. 310926), CD44-BV421 (Cat. 103040), PD1-APC (Cat. 621610) and LAG3-Alexa Fluor 488 (Cat. 369326) and (3) **Tregs panel**: CD3-PECy7 (Cat.317418), CD4-APCCy7 (Cat. 317418), CD25-Alexa Fluor 647 (Cat. 302618), and FOXP3-PE (Cat. 320108).

The staining with each panel of antibodies was performed in 100 μL of fresh anti-coagulated blood sample as previously standardized in our lab (Olexen et al., 2022). For cell surface staining, samples were incubated with each antibody panel on ice for 30 minutes protected from light, and fixed with 100 μL of Citofix Buffer (BD Bioscience) for additional 20 minutes. Then, cells were washed with PBS and centrifuged at 800× *g* for 5 minutes. Afterthat, to eliminate erythrocytes, blood cells were incubated with 1 mL ACK Lysing Buffer (Thermofisher Scientific) for 10 minutes at 25 °C. Then, intracellular staining of FOXP3 was performed only for Tregs characterization employing an specific kit (True-Nuclear™ Transcription Factor Buffer Set, Biolegend). Briefly, 300 μL of 1x True Nuclear Fixation Buffer was added and incubated for 60 min at room temperature protected from light. After that, cell permeabilization was performed by centrifuging the cells at 800× *g* for 5 minutes with 200 μL of the True Nuclear 1x Perm Buffer, repeated twice. The FOXP3 antibody, diluted in True Nuclear 1x Perm Buffer, was added and incubated for 30 min in the dark at room temperature. Cells were maintained with 1x Perm Buffer, and finally, all the three cocktails were washed with 1 mL PBS and analyzed by flow cytometry.

### 2.6 Plasma level of cytokines, chemokines and neurotrophic factors determined by bead-based immunoassay

LEGENDplex (BioLegend) customized Human Inflammation Panel 1 (Cat. 740809) was employed to measure (IL-1β, IFNγ, IL-17, IL-33, IL-8, IL-10, IL-12p70 and IL-23) and the LEGENDplex Human Neuroinflammation Panel 1 (Cat. 740796) for (TGF-β, β-NGF, CX3CL1, BDNF, sTREM-2, IL-18, IL-6, TNFα and MCP-1) in 50 μL of plasma samples (Supplementary Table S2). Briefly, the concentration of each molecule is calculated based on the standard curve and the mean fluorescence intensity of the PE channel for each analyte (Daray et al., 2024a). Additionally, IL-6 was determined by a high-sensitivity ELISA kit (Enzo Life Sciences). All experiments were performed following the manufacturer's instructions.

### 2.7 Analysis by flow cytometry

The samples were run in an external FACS core facility from the National System of Flow Cytometry, Argentina (FACS Canto I, Becton Dickinson). Data were analyzed using Flowjo software (Tree Star Inc).

### 2.8 Data analysis

Descriptive statistics were used to summarize participant characteristics. Categorical variables were reported as absolute and relative percentage frequencies (%), and quantitative variables were reported as means and standard deviations (SD) for normal variables or as median and interquartile range (IQR) for non-normal variables. The Shapiro-Wilk test was used to evaluate whether each quantitative variable had a normal distribution.

Comparisons between group participants were made according to the type of variable. Specifically, categorical variables were compared using Pearson's chi-square test or Fisher's exact test, normal quantitative variables were compared using the ANOVA test, and non-normal quantitative variables were compared using the Kruskal-Wallis rank sum test. In those variables with a significant difference between groups, pairwise comparisons were made using the Holm-Bonferroni adjustment.

Multinomial logistic regression models were fitted to evaluate the association between SI/SA, inflammation, and depressive symptoms. The coefficients were calculated to compare the two groups with current and lifetime suicide ideation or behavior (SI/SA) against the control group. Odds ratios (OR) for each term were evaluated and their corresponding 95% confidence intervals were calculated.

Significance was defined as p < 0.05 for all analyses. Statistical analyses were conducted using RStudio version 2022.02.1 + 461.

## 3 Results

### 3.1 Sociodemographic and clinical characteristics of the participants

Table 1 presents the sociodemographic and clinical characteristics of 105 participants included in the study: 21 with current SI/SA (20.0%), 42 with lifetime SI/SA (40.0%), and 42 participants HC (40.0%). There were no significant differences between the groups regarding sex and age. However, as expected, patients exhibited higher rates of unemployment and lower educational attainment compared to HC. Additionally, patients had higher scores on the Hamilton Depression Rating Scale (HAMD-17), reported a greater number of adverse childhood experiences (ACEs) and stressful life events, and over 85% were receiving psychopharmacological treatment.

**Table 1:** Sociodemographic and clinical characteristics of participants.

**Variable**	**Current SI/SA**	**Lifetime SI/SA**	**Healthy** c**ontrol**	**p-value**
n	21	42	42	
Age (median [IQR])	37.00 [30.00, 44.00]	43.00 [31.75, 52.75]	39.00 [30.25, 49.00]	0.186
Gender = Male (%)	7 (33.3)	11 (26.2)	14 (33.3)	0.738
Civil status (%)				0.092
Married/Living with a partner	6 (28.6)	7 (16.7)	13 (31.0)	
Separated/Divorced/Widower	1 (4.8)	12 (28.6)	5 (11.9)	
Single	14 (66.7)	23 (54.8)	24 (57.1)	
Scholarship (%)				**0.002**
Incomplete high school	6 (28.6)	7 (16.7)	1 (2.4)	
Complete high school	4 (19.0)	7 (16.7)	2 (4.8)	
Incomplete college	5 (23.8)	13 (31.0)	16 (38.1)	
Complete college	4 (19.0)	13 (31.0)	23 (54.8)	
Employment status = Unemployed/Retired (%)	14 (66.7)	15 (35.7)	5 (11.9)	**<0.001**
Diagnostic summary (%)				**<0.001**
Bipolar disorder I	4 (19.0)	16 (38.1)	0 (0.0)	
Bipolar disorder II	5 (23.8)	6 (14.3)	0 (0.0)	
Major depressive disorder	12 (57.1)	20 (47.6)	0 (0.0)	
None depressive disorder	0 (0.0)	0 (0.0)	42 (100.0)	
HAM-D Total score (median [IQR])	15.00 [11.00, 18.00]	5.00 [3.00, 10.00]	0.00 [0.00, 1.00]	**<0.001**
On pshychopharmacological treatment = Yes (%)	18 (85.7)	35 (85.4)	0 (0.0)	**<0.001**
Total number of ACEs (median [IQR])	5.00 [2.00, 6.00]	4.50 [2.00, 6.00]	0.00 [0.00, 1.75]	**<0.001**
IPAQ (%)				0.695
Low	13 (61.9)	24 (57.1)	19 ( 45.2)	
Median	4 (19.0)	8 (19.0)	12 ( 28.6)	
High	4 (19.0)	10 (23.8)	11 ( 26.2)	
Number of Stressful Life Events (median [IQR])	9.00 [7.00, 13.00]	10.50 [8.00, 14.00]	8.50 [6.00, 12.00]	0.121
Stressful Life Events Total Score (median [IQR])	275.00 [206.00, 435.00]	330.50 [218.75, 428.75]	187.50 [139.25, 316.50]	**0.001**
Weight (median [IQR])	70.00 [61.00, 84.00]	67.55 [60.50, 80.00]	70.00 [60.75, 78.50]	0.989
Height (mean (SD))	166.76 (7.81)	163.90 (9.41)	167.76 (8.73)	0.129
BMI (median [IQR])	24.60 [23.50, 30.40]	25.60 [22.68, 30.61]	24.25 [22.00, 27.67]	0.326
Variable	Current SI/SA	Lifetime SI/SA	Healthy Control	p-value
Amount of cigarettes per day (median [IQR])	10.00 [0.00, 20.00]	0.00 [0.00, 3.75]	0.00 [0.00, 0.00]	**<0.001**

Ref.: Current SI/SA: suicide ideation or attempt in the last month. Lifetime SI/SA: history of suicide ideation or attempt before the previous month. HAM-D: Hamilton Depression Rating Scale. ACEs: The Adverse Childhood Experiences questionnaire. IPAQ: International Physical Activity Questionnaire. BMI: Body Mass Index.

### 3.2 Peripheral immunological alterations in patients with current and lifetime SI/SA

**Fig. 1:**
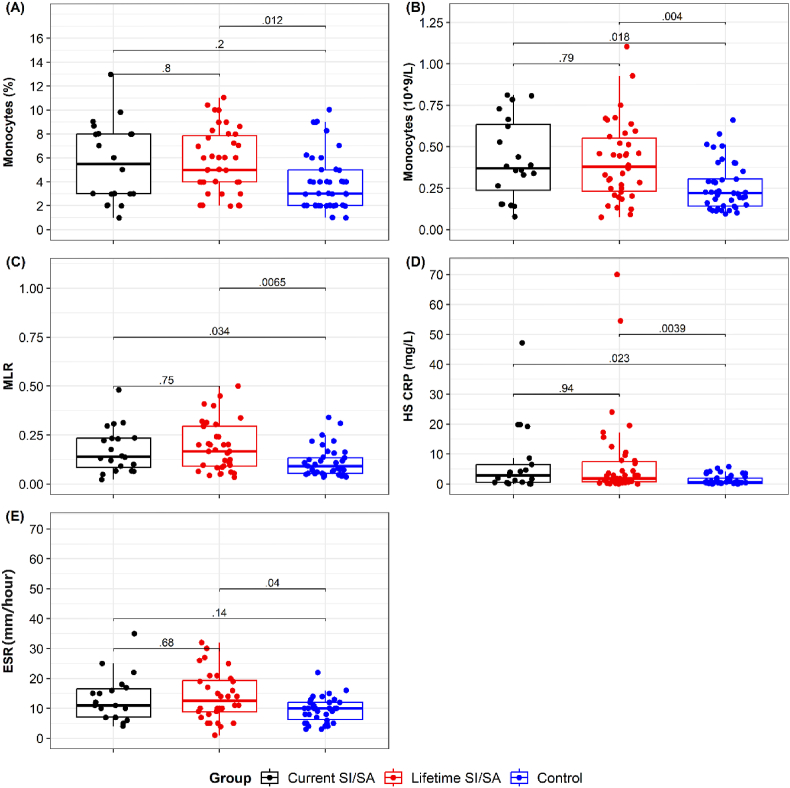
**Comparative analysis of monocyte values, MLR, hs-CRP and ESR among groups.** **A-E.** Independent data showing the percentage of monocytes **(A)**, absolute count of monocytes **(B)**, Monocyte/Lymphocyte ratio, MLR **(C)**, High sensitivity C-reactive protein, HS CRP **(D)** and the eritrosedimentation rate (ESR) **(E)**. Current SI/SA: suicide ideation or attempt in the last month. Lifetime SI/SA: history of suicide ideation or attempt before the previous month. HC: healthy control. Statistical differences among groups were calculated by pairwise comparisons using the Holm-Bonferroni adjustment. The p-value is shown for each comparison.

Significant differences were observed when comparing the HC group with the patients groups. These differences were observed in the median percentage of monocytes (p = 0.016) (Supplementary Table S1), mainly showing an increase in patients with Lifetime SI/SA compared to HC (p = 0.012) (Fig. 1). Additionally, differences were observed in the absolute number of monocytes (p = 0.002) (Supplementary Table S1), being higher in the Current SI/SA group (p = 0.018), as well as in the Lifetime SI/SA group (p = 0.004) compared to HC (Fig. 1). Furthermore, variations were evident in the monocyte/lymphocyte ratio (MLR) (p = 0.004) (Supplementary Table S1), notably increasing in the Current SI/SA group (p = 0.034) (Fig. 1) and Lifetime SI/SA group (p = 0.006) compared to HC (Fig. 1).

Two additional peripheral blood inflammatory markers, hs-CRP and ESR, showed significant increases. We observed notable differences among the groups in hs-CRP concentration (p = 0.002) (Supplementary Table S1), specifically increasing in the Current SI/SA (p = 0.023) and Lifetime SI/SA (p = 0.039) groups compared to HC (Fig. 1). The ESR value also showed a significant difference (p = 0.029) (Supplementary Table S1), being higher in Lifetime SI/SA (p = 0.04) compared to HC.

#### 3.2.1 The cellular inflammatory status of patients with SI/SA is characterized by an altered proportion of monocyte subsets and exhausted CD4 lymphocytes

**Fig. 2:**
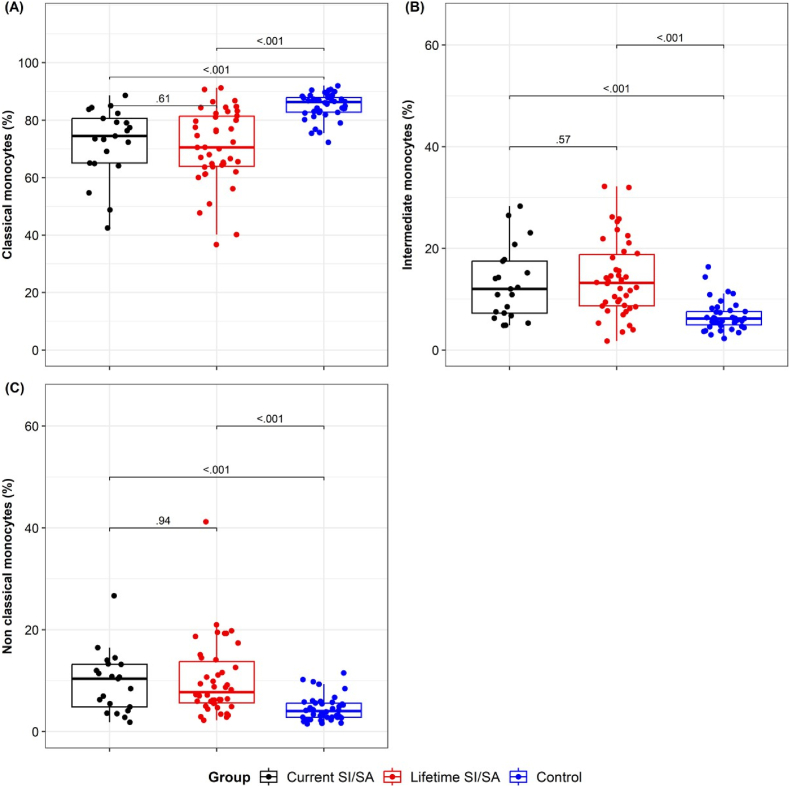
**Comparative analysis of monocyte subsets among groups.** **A-C.** Independent data showing the percentage of classical (CD14^++^CD16^-^) **(A),** intermediate (CD14^+^CD16^+^) **(B)**, and nonclassical (CD14^-^CD16^++^) monocytes **(C)**, determined by flow cytometry. The median and interquartile range of each group is shown. Current SI/SA: suicide ideation or attempt in the last month. Lifetime SI/SA: history of suicide ideation or attempt before the previous month. HC: healthy control. Statistical differences among groups were calculated by pairwise comparisons using the Holm-Bonferroni adjustment. The p-value is shown for each comparison.

The proportion of the three subtypes of circulating monocytes classical (CD14^++^CD16^−^), intermediate (CD14^+^CD16^+^), and nonclassical (CD14^-^CD16^++^) was obtained by flow cytometry (Table 2). A statistically significant difference in the medians per group was observed for the three types of monocytes (p < 0.001 in all three cases), as we previously reported using peripheral blood mononuclear cells (PBMCs) (Nowak et al., 2019). We reinforced these findings with an independent new recruited population of patients employing a direct staining on fresh peripheral blood. We found a higher proportion of nonclassical and intermediate monocytes in concordance with a reduced percentage of classical monocytes in both patients groups vs. HC (Fig. 2).

**Table 2:** Comparison of classical, intermediate, and nonclassical monocytes among groups.

Variable	Current SI/SA	Lifetime SI/SA	Healthy Control	p-value
Classical monocytes (median [IQR])	74.50 [65.10, 80.60]	70.55 [63.95, 81.40]	86.30 [82.80, 87.88]	**<0.001**
Intermediate monocytes (median [IQR])	12.00 [7.25, 17.50]	13.20 [8.69, 18.80]	6.18 [4.94, 7.54]	**<0.001**
Nonclassical monocytes (median [IQR])	10.40 [4.82, 13.20]	7.73 [5.62, 13.72]	4.00 [2.79, 5.55]	**<0.001**

Ref.: Current SI/SA: suicide ideation or attempt in the last month. Lifetime SI/SA: history of suicide ideation or attempt before the previous month. Monocyte subsets are determined by direct immunostaining in peripheral blood using flow cytometry and expressed as a percentage. The three monocyte subsets were defined by the expression levels of CD16 vs. CD14 as classical (CD16^-^CD14^++^), nonclassical (CD16^++^CD14^-^), and intermediate (CD16^+^CD14^+^) as previously reported (Nowak et al., 2019).

**Fig. 3:**
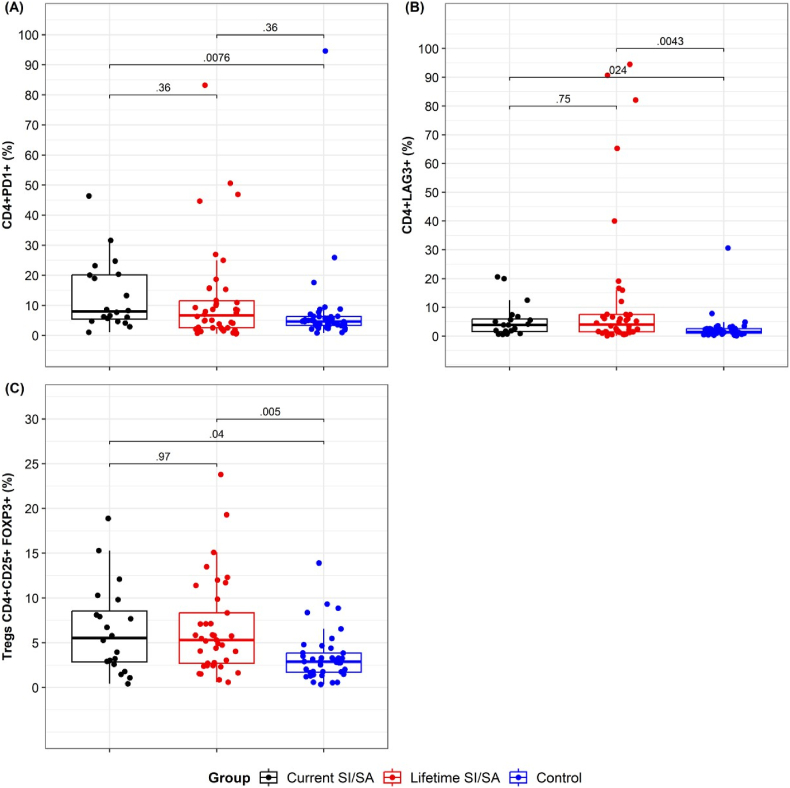
**Comparative analysis of CD4^+^ lymphocyte subsets among groups.** **A-C.** Independent data showing the percentage of PD1^**+**^**(A)** and LAG3^+^**(B)** on CD4^+^ lymphocytes and the percentage of CD4^+^CD25^+^FOXP3^+^ regulatory T cells (Tregs) **(C)** determined by flow cytometry. The median and interquartile range of each group is shown. Current SI/SA: suicide ideation or attempt in the last month. Lifetime SI/SA: history of suicide ideation or attempt before the previous month. HC: healthy control. Statistical differences among groups were calculated by pairwise comparisons using the Holm- Bonferroni adjustment. The *p*-value is shown for each comparison.

While the percentage of CD4 and CD8, and the ratio of CD4/CD8 T lymphocytes, measured by flow cytometry, did not show significant differences among groups, we observed significant changes in the exhaustion markers of CD4^+^ T lymphocytes (Table 3). We found a significant increase in the median percentages of exhaustion marker PD1^+^ (p = 0.0076) in Current SI/SA compared to HC (Fig. 3). Furthermore, we found a significant increase in the frequency of LAG3^+^ in Current SI/SA (p = 0.024) and Lifetime SI/SA (p = 0.004) compared with HC and in the frequency of CD4^+^CD25^+^FOXP3^+^ Tregs in Current SI/SA (p = 0.04) as well as in Lifetime SI/SA (p = 0.005) compared with HC (Fig. 3).

**Table 3:** Comparison of CD4^+^ T lymphocyte subsets among groups.

Variable	Current SI/SA	Lifetime SI/SA	Healthy control	p-value
CD3^+^CD4^+^ (median [IQR])	59.20 [51.80, 64.60]	57.85 [48.95, 62.85]	60.25 [54.05, 65.83]	0.680
CD3^+^CD8^+^ (median [IQR])	28.20 [23.60, 34.50]	27.20 [23.60, 31.70]	28.55 [26.02, 32.17]	0.810
Ratio CD4/CD8 (median [IQR])	1.92 [1.70, 2.64]	2.01 [1.49, 2.61]	2.05 [1.78, 2.43]	0.852
CD4^+^CD44^+^ (median [IQR])	51.10 [38.10, 55.60]	49.55 [30.63, 67.48]	42.00 [32.25, 60.78]	0.849
CD4^+^CD69^+^ (median [IQR])	3.24 [2.09, 10.50]	2.80 [1.32, 7.47]	2.08 [0.90, 3.05]	0.054
CD4^+^PD1^+^ (median [IQR])	8.00 [5.42, 20.18]	6.70 [2.53, 11.52]	4.66 [3.34, 6.27]	**0.023**
CD4^+^LAG3^+^ (median [IQR])	3.83 [1.56, 5.94]	4.03 [1.49, 7.52]	1.43 [1.00, 2.58]	**0.002**
CD4^+^CD25^+^ FOXP3^+^ Tregs (median [IQR])	5.53 [2.85, 8.54]	5.32 [2.70, 8.33]	2.89 [1.71, 3.86]	**0.004**

Ref.: Current SI/SA: suicide ideation or attempt in the last month. Lifetime SI/SA: history of suicide ideation or attempt before the previous month. Lymphocyte subsets are determined by direct immunostaining in peripheral blood using flow cytometry and expressed as a percentage. The activation status of CD3^+^CD4^+^ lymphocytes was assessed by the expression levels of CD69, CD44, PD1, and LAG3. Finally, the frequency of Tregs was determined by CD3^+^CD4^+^CD25^+^FOXP3^+^ cells.

#### 3.2.2 The humoral inflammatory status of patients is characterized by an altered level of sTREM2

**Fig. 4:**
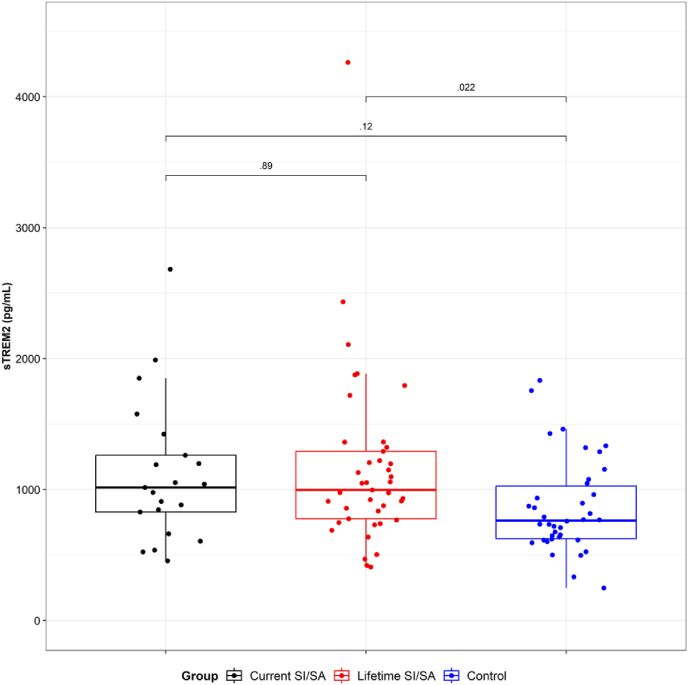
**Comparative analysis of sTREM2 values among groups.** Independent data showing the median of sTREM2, determined in plasma using a customized LEGENDPlex system Human Neuroinflammation Panel. Current SI/SA: suicide ideation or attempt in the last month. Lifetime SI/SA: history of suicide ideation or attempt before the previous month. HC: healthy control. Statistical differences among groups were calculated by pairwise comparisons using the Holm- Bonferroni adjustment. The p-value is shown for each comparison.

We have assessed sixteen plasma markers, including cytokines, chemokines, and neurotrophic factors, employing a customized LEGENDPlex panel. The only group differences were observed in the soluble form of the triggering receptor expressed on myeloid cells 2 (sTREM2) levels (Supplementary Table S2). We have found a robust and significantly higher level of sTREM2, a biomarker of microglia activation, in Lifetime SI/SA compared with HC (p = 0.022) (Fig. 4).

### 3.3 Exploring the role of depressive symptoms in the relationship between immunological alterations and SI/SA

A multinomial logistic regression model was fitted in three steps to examine the relationship between immunological alterations and SI/SA, considering depressive symptoms as a potential moderator. The group was the outcome variable, and hs-CRP and MLR were independent variables used as a proxy for cellular and humoral inflammation. Furthermore, the HAM-D score was considered a possible moderator of the effect of inflammation (hs-CRP and/or MLR) on the SI/SA, so the model included its independent effect and corresponding interactions. Potential confounding variables were also considered: sex, age, and BMI. Healthy control was considered the reference group. The 97 subjects with complete data on all variables were used for adjustment.

In the first step, the initial Multinomial Logistic Regression model (Supplementary Table S3) showed that sex, age, BMI, and interactions between inflammation variables and HAM- D scale were not significant. HAM-D scale and MLR presented significant effects when comparing the two groups of suicidal patients against the HC. However, MLR presented estimates with large standard errors. The second step removed these non-significant variables (Supplementary Table S4), and a step-by-step procedure based on Akaike measure (AIC) was conducted to find the model with the best fit. In the third step of the analysis, the model (Table 4), which included only hs-CRP and HAM-D variables, showed that these factors have independent and additive effects on SI/SA. Specifically, for each one-unit increase in hs-CRP among individuals with the same HAM-D score, the likelihood of having Current SI/SA instead of being a HC increased by 30.7% (OR = 1.307; 95% CI = 1.015–1.686).

**Table 4:** Adjusted multinomial logistic regression.

95% CI							
Group	Variable	Coefficient	Std error	p-value	OR	LL	UL
Current SI/SA	(Intercept)	−5.595	0.932	**< 0.001**	0.004	0.001	0.023
	`HS CRP'	0.268	0.129	**0.038**	1.308	1.015	1.686
	`HAM-D Total score'	1.248	0.278	**< 0.001**	3.484	2.022	6.002
Lifetime SI/SA	(Intercept)	−2.842	0.616	**< 0.001**	0.058	0.017	0.195
	`HS CRP'	0.286	0.128	**0.025**	1.331	1.036	1.709
	`HAM-D Total score'	1.031	0.27	**< 0.001**	2.803	1.651	4.76

Ref.: Healthy Control was used as a reference. Current SI/SA: suicide ideation or attempt in the last month. Lifetime SI/SA: history of suicide ideation or attempt before the previous month. HAM-D: Hamilton Depression Rating Scale. HS CRP: High Sensitivity C-Reactive Protein.

Additionally, when comparing two individuals with identical hs-CRP values and a one-unit difference on the HAM-D scale, the probability of having Current SI/SA instead of being a HC was 3.483 times higher in the individual with the higher HAM-D score (OR = 3.483; 95% CI = 2.022–6.002). Moreover, for each one-unit increase in hs-CRP among individuals with the same HAM-D score, the chance of having Lifetime SI/SA instead of being a HC increased by 33.1% (OR = 1.331; 95% CI = 1.036–1.709). Similarly, when comparing two individuals with the same hs-CRP value and a one-unit difference on the HAM-D scale, the probability of having Lifetime SI/SA instead of being a HC was 2.804 times greater in the subject with the highest HAM-D score (OR = 2.804; 95% CI = 1.651–4.760).

## 4 Discussion

The results of this study further support the concept of dysregulated immune-inflammatory responses in individuals with SI/SA. A key finding is the increased monocytosis characterized by an expansion of intermediate and nonclassical monocyte subsets alongside a heightened MLR in current and lifetime SI/SA patients. Additionally, we observed an elevated frequency of CD4^+^LAG3^+^ lymphocytes and CD4^+^CD25^+^FOXP3^+^ regulatory T cells (Tregs) in SI/SA patients, suggesting a potential compensatory anti-inflammatory response. Another significant observation is the increase of exhaustion markers in CD4^+^ lymphocytes in patients with current SI/SA, a novel finding in these patients. Furthermore, we identified elevated levels of sTREM2, a soluble marker of neuroinflammation, in SI/SA patients for the first time. Additionally, our results indicate that inflammation may play a role in predisposing individuals to SI/SA. The relationship between inflammation and SI/SA is not moderated by depressive symptoms; instead, these factors exhibit independent and additive effects on SI/SA.

In the last few years, accumulating evidence has highlighted the pivotal role of the immune system dysregulation and inflammation in SI/SA (Neupane et al., 2023). The proposed role of inflammation in suicide pathophysiology underscores the direct impact of pro-inflammatory cytokines on neuronal function. Specifically, cytokines like interleukin-1 beta (IL-1β) and TNF-α have been shown to heighten neuronal excitability and diminish synaptic plasticity in the hippocampus, a critical brain region for mood regulation and cognitive function (Serafini et al., 2023). These alterations may induce mood swings, impaired decision-making, and an increased risk of suicidal thoughts and behaviors. Moreover, inflammatory cytokines can compromise the integrity of the blood-brain barrier (BBB), facilitating enhanced infiltration of immune cells and inflammatory mediators into the brain (Courtet et al., 2016). This cascade intensifies neuroinflammation, contributing to both neuronal dysfunction and behavioral changes. Based on this, immunological biomarkers have emerged as promising candidates for evaluating individuals at risk of suicide. Significant immune alterations in inflammatory biomarkers have been observed in blood, cerebrospinal fluid, and postmortem brain samples of individuals with SI/SA (Chang et al., 2016; Keaton et al., 2019; Sudol and Mann, 2017; Ducasse et al., 2015; Courtet et al., 2015; Serafini et al., 2020; González-Castro et al., 2021). A recent meta-analysis has further supported these findings, showing that these changes are independent of their association with major psychiatric disorders and primarily indicative of an individual's current SA (Neupane et al., 2023).

Most of these studies have a cross-sectional design and focus mainly on the immune system's humoral component, with few studies focused on the cellular immune response (Nowak et al., 2019). This leads us to consider the following questions: Are specific alterations in cell-mediated immune response associated with SI/SA? Is the relationship between immune system and SI/SA moderate by depressive symptoms?

The cellular immune response comprises both innate and adaptive components. Monocytes play a pivotal role in the innate immune system, while T lymphocytes are key components of the adaptive immune system. These cells types play essential and enduring roles in immune surveillance within the peripheral and central nervous systems (Schiweck et al., 2020b; Alvarez-Mon et al., 2019). Given the significant bidirectional communication between peripheral and cerebral immune cells, peripheral immune cells have the potential to serve as surrogate markers for CNS processes (Prinz and Priller, 2017). Our research group was the first to notice that patients with SA and MDD exhibited significantly elevated levels of pro-inflammatory cytokines IL-12 and IL-6 in their plasma and that these cytokines were associated with an increased number of nonclassical (CD16^++^CD14^−^) monocytes and a higher activation state of classical monocytes (CD16^-^CD14^+^ (Nowak et al., 2019). These observations were then replicated by another research group (Alvarez-Mon et al., 2019; Lynall et al., 2020). In the present case-control study we observed a higher proportion of nonclassical and intermediate monocytes and a concomitant reduced percentage of classical monocytes in concordance with the transitional activation model described by Patel et al. (2017) in both current and lifetime SI/SA patients compared to HC individuals. These results suggest that monocyte proportion changes could be trait markers rather than state markers. However, longitudinal and prospective studies are crucial to better understand the dynamics of these changes in the same individuals with and without SI/SA.

A practical approach for assessing leukocyte imbalance on SI/SA in clinical practice involves using cell ratios measured in the hemogram (Velasco et al., 2023). Neutrophil–lymphocyte ratio (NLR), monocyte-lymphocyte ratio (MLR), and platelet-lymphocyte ratio (PLR) are considered biomarkers of systemic inflammation (Velasco et al., 2023) and are associated with more severe depression and elevated suicide risk (SR) (Puangsri and Ninla-Aesong, 2021; Zheng et al., 2022). Specifically, a higher MLR has been suggested to serve as a more predictive biomarker for suicide attempts in adolescents with MDD than either NLR or PLR (Puangsri and Ninla-Aesong, 2021). A recent systematic review and meta-analysis showed increased MLR in depressed individuals with SI/SA compared to those without SI/SA (Daray et al., 2024b). Consistent with these findings, our study showed a significant increase in MLR in patients with SI/SA. An elevated MLR is associated with an overexpression of inflammatory cytokines related to activated monocytes and, as consequence, this activation triggers microglia in the brain, causing neuroinflammation (Zheng et al., 2022). Thus, MLR could serve as an attractive and convenient trait marker of suicidal vulnerability in patients with AD.

Regarding T cell lineage, a recent study reveals that MDD patients with suicidal behavior (SB) exhibit alterations in circulating T helper cells, with notably higher Th17 cells when compared to depressed patients without SB (Schiweck et al., 2020b). However, this study has a limitation, as they assess immune factors individually, and a more comprehensive investigation encompassing multiple factors and their interplay is warranted. Our present case-control study reveals that adaptive immunity is also altered in SI/SA patients. While the total number of lymphocytes remains similar between the groups, the function of these cells is affected in patients with SI/SA. A significant increase in the frequency of CD4^+^CD25^+^FOXP3^+^ regulatory T cells (Tregs) was observed in both patient groups compared to HC. Tregs play a pivotal role in maintaining immune homeostasis and are believed to be involved in the dysregulation of immune mechanisms (Carvalho et al., 2020; Maes and Carvalho, 2018). The increase in Tregs percentage suggests a compensatory immune mechanism to counterbalance potential proinflammatory processes and regulate immune activity.

We also observed a substantial increase in the median percentages of exhaustion markers, such as Programmed cell death protein1 (PD1) and lymphocyte activation gene 3 (LAG3), suggesting for the first time T cell exhaustion in SI/SA patients. Additionally, PD1 shows significant differences between current SI/SA and HC, potentially indicating a temporary state (state marker), whereas LAG3 is increased in both current and lifetime SI/SA compared to HC, suggesting a trait marker. Longitudinal studies tracking these immune changes over time are essential for comprehensively understanding these findings. PD1 (Filippone et al., 2022) and LAG3 (Maruhashi and Sugiura, 2020) are inhibitory receptors (usually known as checkpoints) highly expressed by exhausted cells. Tcell exhaustion occurs due to continuous antigen stimulation in the tumor microenvironment or in chronic viral infections and is characterized by progressive Tcell dysfunction (Xie et al., 2023). However, there is no report of these markers in individuals with psychiatric disorders or SI/SA. Investigating Tcell exhaustion in patients with SI/SA is a groundbreaking development in our field, and it holds the promise of opening new avenues for therapeutic interventions. The discovery of checkpoint inhibitors substantially improved the course of some types of cancer (e.g., melanoma) and infections (e.g., hepatitis B). Understanding the parallels between T cell exhaustion in SI/SA and immune responses in other health conditions could potentially address innovative treatment strategies to transform the care landscape for individuals with SI/SA.

Our study has also uncovered a potential novel and likely more specific biomarker for neuroinflammation in individuals with lifetime SI/SA, the soluble triggering receptor expressed on myeloid cell 2 (sTREM2). This protein receptor is primarily expressed in microglial cells within the brain and plays a crucial role in microglial activation, survival, and apoptosis (Wang et al., 2024). Alterations in sTREM2 levels have been linked to microglial activation in neurodegenerative and neuroinflammatory diseases (Wang et al., 2024; Guo et al., 2022). The potential involvement of sTREM2 in SI/SA remains largely unexplored, and our current study represents the first instance of reporting elevated sTREM2 levels in the plasma of individuals with lifetime SI/SA compared to healthy controls. This discovery suggests a potential pathological process occurring in microglial cells, opening up a novel avenue for investigation in the field of suicidal behavior.

Inflammation has been consistently linked to depressive symptoms and suicidal SI/SA, suggesting that inflammatory processes may contribute to SI/SA beyond what is associated with depressive symptoms. A recent meta-analysis has demonstrated that proinflammatory changes in individuals with SB are independent of their association with major psychiatric disorders and primarily reflect an individual's current suicidal state (Neupane et al., 2023). However, many studies failed to adequately account for the potential role of depressive symptoms as a mediator or moderator of the relationship between inflammation and SI/SA (Serafini et al., 2020). The results of our multinomial analysis suggest a significant relationship between SI/SA, immunological alteration, and depressive symptoms. First, we found that hs-CRP, a marker of inflammation, is associated with current and lifetime SI/SA, which suggests that inflammation may play an important role in predisposing to SI/SA. Secondly, we observed that depressive symptoms, measured by the HAM-D scale, are also related to current and lifetime SI/SA. These findings underscore that these factors exert independent and cumulative effects on SI/SA. These findings have potential therapeutic implications, suggesting that for patients with SI/SA addressing inflammation may be necessary beyond treating previously identified depressive symptoms.

This study's strength stands in its multicenter recruitment, rigorous patient selection process (excluding individuals with known causes of inflammation or immune system activation), and comprehensive analysis of immune cells. However, some limitations should be noted. The cross-sectional design provides only a snapshot of individuals with SI/SA; nevertheless, it is a necessary approach for generating new hypotheses. While the overall sample size was moderate, the size of each group for comparisons might have been insufficient, potentially leading to the non-detection of significant differences among the patient groups. Another limitation is that the majority of participants were undergoing psychopharmacological treatment at the time of inclusion. It is widely recognized that various psychotropic medications can influence the immune system, thereby introducing a potential confounding factor in the interpretation of immunological findings. Despite these limitations, our study contributes valuable insights into the immunological aspects of SI/SA, offering a comprehensive examination of both the humoral and the innate and adaptive cellular components. To comprehensively understand the implications of these immune alterations, further research is essential, particularly with longitudinal studies. This will pave the way for potential advancements in clinical practice and a more thorough understanding of the dynamics involved in SI/SA.

## Funding

Grants from supported the study:

1) Brain & Behavior Research Foundation. 2019 NARSAD Young Investigator Grant ID 27855 to F.M.D.
2) Ministerio de Ciencia, Tecnología e Innovación, Argentina through PID-2018-0054 to F.M.D.
3) Consejo Nacional de Investigaciones Científicas y Técnicas (CONICET), Argentina, trough PIP 2015-0567 to A.E.E.
4) Agencia Nacional de Promoción Científica y Tecnológica (ANPCyT), Argentina, through PICT 2017-2431 to A.E.E and PICT 2018-03070 to E.A.C.S.

A.R.A., and L.C.C are recipients of fellowships from CONICET, J.R. is a recipient of a fellowship from Ministry of Health, Autonomous City of Buenos Aires, and V.T., A.O. and R.I.A.C are recipients of fellowships from the Agencia Nacional de Promoción Científica y Tecnológica (ANPCyT), Argentina.

F.M.D., E.A.C.S, and A.E.E. are career investigators at CONICET, Argentina.

L.G. is a career investigator in the Health Researcher Career at the Ministry of Health, Autonomous City of Buenos Aires, Argentina.

The funding sources had no involvement in the study design, in the collection, analysis, and interpretation of data, in the writing of the manuscript, and in the decision to submit the paper for publication.

## CRediT authorship contribution statement

**Leandro Nicolás Grendas:** Investigation, Writing – original draft, Writing – review & editing. **Eugenio Antonio Carrera Silva:** Funding acquisition, Investigation, Methodology, Writing – review & editing. **Romina Isabel Álvarez Casiani:** Investigation, Writing – review & editing. **Alejandro Olaviaga:** Investigation, Writing – review & editing. **Josefina Robetto:** Investigation, Writing – review & editing. **Ángeles Romina Arena:** Formal analysis, Validation, Visualization, Writing – review & editing. **Vera Tifner:** Formal analysis, Investigation, Visualization, Writing – review & editing. **Luciana Carla Chiapella:** Data curation, Formal analysis, Writing – review & editing. **Marcelo Fisichella:** Investigation. **Melina Bianca Penna:** Investigation, Writing – review & editing. **Fernando Hunter:** Investigation, Writing – review & editing. **Cintia Romina Prokopez:** Investigation, Writing – review & editing. **Andrea Emilse Errasti:** Funding acquisition, Investigation, Methodology, Writing – review & editing. **Federico Manuel Daray:** Conceptualization, Funding acquisition, Methodology, Supervision, Writing – review & editing.

## Declaration of competing interest

None.
