# Supplementary material for: Immunological alterations in patients with current and lifetime suicide ideation and attempts: Examining the relationship with depressive symptoms
Source: Brain Behav Immun Health. 2024 Apr 25;38:100777. doi: 10.1016/j.bbih.2024.100777 (PMC11067476; doi:10.1016/j.bbih.2024.100777)
Supplement: Multimedia component 1 [file mmc1.docx]

**Supplementary Table S1**: Comparison of biochemical parameters among groups.

| **Variable** | **Current SI/SA** | **Lifetime SI/SA** | **Healthy Control** | **p-value** |
| --- | --- | --- | --- | --- |
| Hematocrit (median [IQR]) | 41.45 [39.58, 44.00] | 41.20 [37.80, 43.70] | 42.50 [40.90, 44.30] | 0.439 |
| Hemoglobin (median [IQR]) | 14.05 [12.90, 14.80] | 13.35 [12.47, 14.40] | 14.10 [13.40, 14.70] | 0.063 |
| Erythrocytes (mean (SD)) | 4.78 (0.55) | 4.64 (0.48) | 4.85 (0.40) | 0.112 |
| MCV (median [IQR]) | 90.00 [86.10, 92.19] | 88.73 [85.37, 91.63] | 86.47 [84.06, 89.54] | **0.045** |
| MCH (median [IQR]) | 29.67 [28.55, 30.34] | 28.87 [27.80, 29.95] | 28.79 [27.86, 29.71] | 0.403 |
| MCHC (median [IQR]) | 32.91 [32.70, 33.50] | 32.65 [32.10, 33.48] | 33.33 [32.51, 33.96] | 0.051 |
| Leukocytes (median [IQR]) | 7.55 [6.50, 9.27] | 6.75 [5.45, 8.84] | 6.60 [5.70, 7.50] | 0.222 |
| Segmented neutrophils Percentage (median [IQR]) | 57.65 [50.75, 63.50] | 58.00 [50.90, 65.50] | 55.00 [50.00, 60.00] | 0.339 |
| Segmented neutrophils absolute count (median [IQR]) | 4.36 [3.30, 5.57] | 3.84 [2.83, 5.08] | 3.59 [3.02, 4.42] | 0.249 |
| Lymphocytes Percentage (mean (SD)) | 34.67 (10.52) | 34.38 (10.53) | 38.16 (6.83) | 0.121 |
| Lymphocytes absolute count (median [IQR]) | 2.52 [2.18, 3.03] | 2.12 [1.87, 2.57] | 2.52 [2.15, 2.77] | 0.055 |
| Monocytes Percentage (median [IQR]) | 5.50 [3.00, 8.00] | 5.00 [4.00, 7.85] | 3.00 [2.00, 5.00] | **0.016** |
| Monocytes absolute count (median [IQR]) | 0.37 [0.24, 0.63] | 0.38 [0.23, 0.55] | 0.22 [0.14, 0.30] | **0.002** |
| Eosinophils Percentage (median [IQR]) | 2.00 [1.45, 2.00] | 2.00 [1.85, 3.00] | 2.00 [1.00, 3.00] | 0.741 |
| Eosinophils absolute count (median [IQR]) | 0.14 [0.10, 0.24] | 0.14 [0.10, 0.20] | 0.13 [0.10, 0.20] | 0.856 |
| Basophils Percentage (median [IQR]) | 0.00 [0.00, 0.00] | 0.00 [0.00, 0.23] | 0.00 [0.00, 0.00] | 0.133 |
| Basophils absolute count (median [IQR]) | 0.00 [0.00, 0.00] | 0.00 [0.00, 0.01] | 0.00 [0.00, 0.00] | 0.132 |
| Platelets (median [IQR]) | 219.00 [193.00, 245.00] | 247.00 [236.65, 277.75] | 243.00 [205.00, 292.60] | 0.182 |
| ESR (median [IQR]) | 11.00 [7.00, 16.50] | 14.00 [9.00, 20.00] | 10.00 [6.25, 12.00] | **0.029** |
| Urea (median [IQR]) | 29.00 [24.50, 34.50] | 28.00 [23.50, 34.00] | 29.00 [23.00, 36.00] | 0.947 |
| Creatinine (median [IQR]) | 7.90 [1.00, 9.35] | 7.00 [0.84, 9.55] | 8.10 [7.22, 10.00] | 0.087 |
| GOT (median [IQR]) | 25.50 [17.75, 35.00] | 22.00 [17.50, 29.25] | 25.00 [18.00, 30.00] | 0.747 |
| GPT (median [IQR]) | 24.50 [15.50, 34.75] | 27.50 [15.50, 33.00] | 24.00 [18.00, 31.00] | 0.942 |
| Alkaline phosphatase (median [IQR]) | 116.00 [79.00, 188.50] | 155.00 [87.25, 186.75] | 155.00 [118.00, 179.00] | 0.466 |
| Total Bilirubin (median [IQR]) | 0.50 [0.36, 0.71] | 0.46 [0.32, 0.57] | 0.66 [0.50, 0.86] | **0.002** |
| Sodium (mean (SD)) | 137.16 (2.77) | 138.52 (3.92) | 138.37 (3.21) | 0.247 |
| Potassium (median [IQR]) | 4.28 [4.00, 4.60] | 4.30 [4.00, 4.50] | 4.30 [4.00, 4.50] | 0.923 |
| Chloride (median [IQR]) | 99.00 [91.00, 100.00] | 99.00 [90.00, 102.10] | 93.00 [89.00, 99.00] | 0.169 |
| HS CRP (median [IQR]) | 2.81 [0.51, 6.45] | 1.77 [0.64, 7.51] | 0.55 [0.20, 1.92] | **0.002** |
| NLR (median [IQR]) | 1.85 [1.30, 2.20] | 1.75 [1.24, 2.67] | 1.38 [1.14, 1.76] | 0.089 |
| MLR (median [IQR]) | 0.14 [0.08, 0.23] | 0.17 [0.09, 0.29] | 0.09 [0.05, 0.13] | **0.004** |
| PLR (mean (SD)) | 96.19 (29.86) | 133.05 (42.15) | 131.04 (31.24) | 0.054 |

Ref.: Current SI/SA: suicide ideation or attempt in the last month. Lifetime SI/SA: history of suicide ideation or attempt before the previous month. MCV: mean corpuscular volume.

MCH: mean corpuscular hemoglobin. MCHC: mean corpuscular hemoglobin concentration. ESR: Erythrocyte sedimentation rate. GOT: glutamic-oxaloacetic transaminase. GPT: glutamic pyruvic transaminase. hs-CRP: High Sensitivity C-reactive Protein. NLR: Neutrophil/Lymphocyte ratio. MLR: Monocyte/Lymphocyte ratio. PLR: Platelet/Lymphocyte ratio. Measure Units: Hematocrit (%), Hemoglobin (g/dL), Erythrocytes (10^12^ cells/L), MCV (femtolitres), MCH (picograms/cell), MCHC (g/dL). Leukocytes, Segmented neutrophils, Lymphocytes, Monocytes, Eosinophils, Basophils, and platelets are expressed as 10^9^ cells/L. ESR (mm/hour). Urea and creatinine are in mg/dL. GOT, GPT, and Alkaline phosphatase are in IU/L. Total Bilirubin (mg/dL). Sodium, Potassium, and Chloride are expressed as mmol/L. hs-CRP (mg/L).
