# Supplementary material for: Immunological alterations in patients with current and lifetime suicide ideation and attempts: Examining the relationship with depressive symptoms
Source: Brain Behav Immun Health. 2024 Apr 25;38:100777. doi: 10.1016/j.bbih.2024.100777 (PMC11067476; doi:10.1016/j.bbih.2024.100777)
Supplement: Multimedia component 2 [file mmc2.docx]

**Supplementary Table S2**: Comparison of humoral marker measurements among groups

| **Variable** | **Current SI/SA** | **Lifetime SI/SA** | **Healthy Control** | **p-value** |
| --- | --- | --- | --- | --- |
| MCP1 (median [IQR]) | 52.61 [32.43, 114.55] | 49.28 [33.22, 67.75] | 53.45 [42.12, 66.44] | 0.726 |
| sTREM2 (median [IQR]) | 1016.18 [828.92, 1261.88] | 997.07 [776.37, 1292.27] | 763.70 [626.05, 1026.06] | **0.020** |
| BDNF (median [IQR]) | 4583.05 [2370.96, 7063.01] | 3852.42 [2329.44, 5770.90] | 5063.97 [3295.16, 6908.95] | 0.350 |
| IL-6 (median [IQR]) | 1.41 [0.92, 2.20] | 1.43 [0.93, 2.48] | 1.33 [0.86, 2.00] | 0.921 |
| bNGF (median [IQR]) | 10.24 [6.60, 19.24] | 14.79 [7.66, 19.79] | 9.49 [2.61, 20.06] | 0.319 |
| IL-18 (median [IQR]) | 123.67 [33.55, 226.79] | 135.13 [52.17, 229.72] | 124.25 [71.50, 161.28] | 0.812 |
| TNF-alpha (median [IQR]) | 38.44 [8.72, 74.08] | 43.46 [0.00, 98.90] | 32.65 [0.00, 141.02] | 0.928 |
| CX3CL-1 (median [IQR]) | 597.77 [431.06, 866.87] | 570.04 [426.10, 1434.08] | 667.86 [436.02, 1234.20] | 0.428 |
| IL-1beta (median [IQR]) | 23.24 [12.15, 79.88] | 30.11 [12.59, 56.11] | 18.78 [9.49, 42.38] | 0.578 |
| IFN-gama (median [IQR]) | 11.30 [5.84, 26.10] | 12.71 [8.32, 19.25] | 9.35 [6.02, 15.37] | 0.288 |
| IL-8 (median [IQR]) | 10.61 [4.01, 23.08] | 12.19 [1.27, 52.36] | 12.40 [4.86, 42.65] | 0.842 |
| IL-10 (median [IQR]) | 5.65 [0.80, 11.16] | 6.79 [0.00, 19.59] | 3.97 [0.00, 10.96] | 0.703 |
| IL-12p70 (median [IQR]) | 6.00 [4.36, 9.10] | 6.66 [4.29, 11.82] | 5.86 [2.99, 10.29] | 0.679 |
| IL-17A (median [IQR]) | 1.85 [0.68, 3.75] | 1.92 [0.19, 3.95] | 0.94 [0.00, 2.30] | 0.093 |
| IL-23 (median [IQR]) | 8.67 [3.86, 17.14] | 12.13 [4.03, 19.88] | 6.52 [3.90, 13.89] | 0.590 |
| IL-33 (median [IQR]) | 59.07 [22.21, 94.36] | 56.92 [21.21, 102.89] | 51.44 [8.15, 82.96] | 0.629 |

Ref.: Current SI/SA: suicide ideation or attempt in the last month. Lifetime SI/SA: history of suicide ideation or attempt before the previous month. The LEGENDPlex system was used to assess cytokines (IL-6, IL-18, TNF-alpha, IL-1beta, IFN-gama, IL-10, IL-12p70, IL-17A, IL-23, and IL-33), chemokines (MCP-1: Monocyte Chemoattractant Protein-1, CX3CL-1: Chemokine (C-X3-C motif) ligand 1, and IL-8/CXCL8: Interleukin 8/ chemokine (C-X-C motif) ligand 8) and neurotrophic factors (sTREM2: Soluble Triggering receptor expressed on myeloid cells 2, BDNF: Brain-derived neurotrophic factor and bNGF: Beta Nerve Growth Factor) in the plasma of patients and Healthy controls. All humoral marker concentrations were measured in pg/mL.
