# Supplementary material for: Immunological alterations in patients with current and lifetime suicide ideation and attempts: Examining the relationship with depressive symptoms
Source: Brain Behav Immun Health. 2024 Apr 25;38:100777. doi: 10.1016/j.bbih.2024.100777 (PMC11067476; doi:10.1016/j.bbih.2024.100777)
Supplement: Multimedia component 3 [file mmc3.docx]

**Supplementary Table S3**: Initial Multinomial Logistic Regression Model

|  |  |  |  |  |  |  | **95% CI** |
| --- | --- | --- | --- | --- | --- | --- | --- |
| **Group** | **Variable** | **Coefficient** | **Std error** | **p-value** | **OR** | **LL** | **UL** |
|  | (Intercept) | -4.915 | 3.713 | 0.186 | 0.007 | 0 | 10.623 |
|  | `HS CRP` | 0.168 | 0.249 | 0.5 | 1.183 | 0.726 | 1.927 |
|  | MLR | -7.617 | 1.841 | **< 0.001** | 0 | 0 | 0.018 |
|  | `HAM-D Total score` | 1.571 | 0.524 | **0.003** | 4.813 | 1.724 | 13.438 |
| Current SI/SA | GenderMale | 0.996 | 1.372 | 0.468 | 2.707 | 0.184 | 39.8 |
|  | Age | -0.015 | 0.06 | 0.805 | 0.985 | 0.876 | 1.108 |
|  | BMI | 0.005 | 0.151 | 0.973 | 1.005 | 0.748 | 1.351 |
|  | MLR:`HAM-D Total score` | -0.671 | 2.139 | 0.754 | 0.511 | 0.008 | 33.848 |
|  | `HS CRP`:`HAM-D Total score` | -0.017 | 0.029 | 0.553 | 0.983 | 0.928 | 1.041 |
|  | (Intercept) | -8.63 | 3.479 | **0.013** | 0 | 0 | 0.163 |
|  | `HS CRP` | 0.353 | 0.19 | 0.062 | 1.424 | 0.982 | 2.064 |
|  | MLR | 11.132 | 4.909 | **0.023** | 68331.589 | 4.53 | 1030723888.333 |
|  | `HAM-D Total score` | 1.56 | 0.536 | **0.004** | 4.756 | 1.665 | 13.591 |
| Lifetime SI/SA | GenderMale | 0.417 | 1.074 | 0.698 | 1.518 | 0.185 | 12.469 |
|  | Age | 0.084 | 0.049 | 0.086 | 1.087 | 0.988 | 1.196 |
|  | BMI | 0.009 | 0.138 | 0.949 | 1.009 | 0.77 | 1.322 |
|  | MLR:`HAM-D Total score` | -2.13 | 2.371 | 0.369 | 0.119 | 0.001 | 12.388 |
|  | `HS CRP`:`HAM-D Total score` | -0.031 | 0.027 | 0.247 | 0.97 | 0.92 | 1.022 |

Ref.: Healthy Control was used as a reference. Current SI/SA: suicide ideation or attempt in the last month. Lifetime SI/SA: history of suicide ideation or attempt before the previous month. HAM-D: Hamilton Depression Rating Scale. HS CRP: High Sensitivity C-reactive Protein. MLR: Monocyte/Lymphocyte ratio. BMI: Body Mass Index.
