# Supplementary material for: Immunological alterations in patients with current and lifetime suicide ideation and attempts: Examining the relationship with depressive symptoms
Source: Brain Behav Immun Health. 2024 Apr 25;38:100777. doi: 10.1016/j.bbih.2024.100777 (PMC11067476; doi:10.1016/j.bbih.2024.100777)
Supplement: Multimedia component 4 [file mmc4.docx]

**Supplementary Table S4:** Multinomial Logistic Regression Model without confounding variables.

|  |  |  |  |  |  |  | **95% CI** |
| --- | --- | --- | --- | --- | --- | --- | --- |
| **Group** | **Variable** | **Coefficient** | **Std error** | **p-value** | **OR** | **LL** | **UL** |
| (Intercept) |  | -4.966 | 1.599 | **0.002** | 0.007 | 0 | 0.16 |
| `HS CRP` |  | 0.138 | 0.215 | 0.52 | 1.148 | 0.753 | 1.75 |
| MLR |  | -1.246 | 10.079 | 0.902 | 0.288 | 0 | 109046423.43 |
| Current SI/SA |  |  |  |  |  |  |  |
| `HAM-D Total score` | | 1.395 | 0.472 | **0.003** | 4.034 | 1.599 | 10.176 |
| MLR:`HAM-D Total score` | | -1.109 | 2.33 | 0.634 | 0.33 | 0.003 | 31.761 |
| `HS CRP`:`HAM-D Total score` | | -0.014 | 0.025 | 0.587 | 0.986 | 0.939 | 1.036 |
| (Intercept) |  | -4.569 | 1.167 | **< 0.001** | 0.01 | 0.001 | 0.102 |
| `HS CRP` |  | 0.34 | 0.138 | **0.014** | 1.405 | 1.072 | 1.843 |
| MLR |  | 10.723 | 5.45 | **0.049** | 45391.512 | 1.042 | 1977527652.899 |
| Lifetime SI/SA |  |  |  |  |  |  |  |
| `HAM-D Total score` | | 1.36 | 0.461 | **0.003** | 3.895 | 1.578 | 9.613 |
| MLR:`HAM-D Total score` | | -2.017 | 2.224 | 0.365 | 0.133 | 0.002 | 10.405 |
| `HS CRP`:`HAM-D Total score` | | -0.028 | 0.022 | 0.201 | 0.973 | 0.932 | 1.015 |

Ref.: Healthy Control was used as a reference. Current SI/SA: suicide ideation or attempt in the last month. Lifetime SI/SA: history of suicide ideation or attempt before the previous month. HAM-D: Hamilton Depression Rating Scale. HS CRP: High Sensitivity C-reactive Protein. MLR: Monocyte/Lymphocyte ratio.
